# Localization Algorithms for Hearing Devices Influenced by Individual Variability in Ear Acoustics

**DOI:** 10.3390/biomimetics11070467

**Published:** 2026-07-03

**Authors:** Jakeh E. Orr, Yan Gai

**Affiliations:** Biomedical Engineering, School of Science and Engineering, Saint Louis University, 3507 Lindell Blvd, St. Louis, MO 63103, USA; jakehorr@gmail.com

**Keywords:** sound localization, HRTF, front–back confusion, binaural, hearing aids

## Abstract

Background: Head-related transfer functions (HRTFs) contain time and level cues and may be utilized in automatic algorithms to identify locations of sound, a desirable feature for next-generation hearing devices. Due to substantial variability in individual head sizes and ear acoustics, individualized HRTFs are expected to provide the best localization results. However, acquiring individualized HRTFs for each user is time-consuming. Methods: This study constructed three binaural and/or monaural algorithms suitable for hearing devices. A linear classifier was trained on HRTF databases from a subset of subjects and used to predict sound locations for other individuals to evaluate cross-subject variability. Results: Using the CIPIC Database, a “two-step” method achieved a horizontal localization error of 1.0° and a vertical error of 30.4° sequentially. With the 3D3A Database, the horizontal and vertical errors were 5.6° and 36.5°, respectively. Both datasets yielded improved accuracy when frontal and rear hemifields were simulated separately, with trends remaining consistent across databases. When subjects were grouped by gender, classifiers trained on women’s HRTFs performed well in predicting men’s localization, whereas classifiers trained on men’s HRTFs resulted in significantly larger errors. Conclusions: These findings offer insights into the localization cues embedded in HRTFs and demonstrate the influences of inter-subject variability for spatial hearing devices.

## 1. Introduction

Spatial separations between speech and interfering noise can substantially improve speech intelligibility, a phenomenon known as “spatial release from masking” [1]. The human auditory system achieves this remarkable capability by integrating binaural and spectral cues to segregate competing sound sources within complex acoustic environments. This biological mechanism has become an important source of inspiration for the field of biomimetics, particularly for the development of intelligent auditory prostheses, humanoid robots, neuromorphic acoustic sensors, and spatially aware hearing-assistive devices. Mimicking the human ability to localize and separate sound sources remains one of the major challenges in biomimetic auditory engineering. Conventional hearing aids often degrade or distort spatial hearing cues, thereby reducing users’ abilities to localize sound sources and separate speech from background noise. Consequently, modern hearing-aid systems increasingly seek to restore biologically inspired spatial hearing functions, including directional sensitivity and spatial release from masking. Recent advances in machine learning and auditory artificial intelligence have accelerated the development of adaptive hearing-assistive systems capable of individualized amplification and spatial processing [2]. Furthermore, studies examining extended-wear hearing devices have continued to demonstrate the importance of preserving natural localization cues for spatial perception [3].

Human sound localization relies on multiple acoustic cues generated by the interaction between incoming sound waves and the anatomy of the head and ears. Localization in azimuth primarily depends on interaural time differences (ITDs) and interaural level differences (ILDs) between sounds arriving at the two ears [4]. Localization in elevation relies on monaural spectral cues produced by the filtering characteristics of the pinna. ITDs represent the temporal delay between sound arrivals at the two ears and provide directional information regarding the angular position of a sound source relative to the head. For periodic stimuli, such as pure tones or amplitude-modulated signals, ongoing ITDs are quantified through interaural phase differences (IPDs), in which the waveforms arriving at the two ears are temporally shifted until optimal alignment is achieved [5]. IPDs therefore serve as an effective representation of ITDs for periodic acoustic stimuli [6]. ILDs arise from the acoustic shadowing effects produced by the head and pinnae, which create differences in sound intensity and spectral distribution between the two ears [7].

The duplex theory [8] states that horizontal localization by humans at low frequencies depends on ITDs or IPDs of the sound, whereas ILDs are unreliable at low frequencies. Meanwhile, horizontal localization at high frequencies depends on ILDs, as well as ITDs of the sound envelope (i.e., the instantaneous amplitude fluctuations) [9]. The fact that there are specialized neurons in the medial and lateral superior olives encoding the ITDs and ILDs separately supports the use of this mechanism for mammalian sound localization [4,9,10,11]. Collectively, these binaural and monaural cues are contained in head-related transfer functions (HRTFs), which characterize the acoustic filtering properties of the head, torso, and pinnae [12,13,14]. HRTFs therefore represent a biologically inspired encoding mechanism through which humans achieve three-dimensional spatial hearing. The review by Bruschi et al. [15] on HRTF generation and spatial-audio synthesis has highlighted the importance of biomimetic HRTF modeling for virtual reality, robotic audition, and immersive human–machine interfaces [15].

The present study investigated the feasibility of training machine learning algorithms using HRTFs from existing human databases and subsequently applying the trained models to previously unseen users without recording individualized HRTFs. HRTFs from the Center for Imaging Processing and Integrated Computing (CIPIC) Database [16] and the Princeton 3D3A Laboratory Database [17] were examined separately and used as known inputs for training localization models. By leveraging large-scale human acoustic datasets, the present work aims to establish a biologically inspired framework for generalized auditory spatial perception. The present study developed three novel biomimetic localization strategies based on the HRTFs obtained from the CIPIC and 3D3A Databases. We then evaluated and compared the localization performance of the three methods using a simple linear classifier.

First, “binaural HRTF (bHRTF)” features were constructed by concatenating ITDs/IPDs and ILDs across a broad frequency range. The efficacy of this representation was examined for localization in both azimuth and elevation. Previous studies have shown that localization based solely on ITDs or ILDs is often limited to the frontal hemifield due to front–back confusion, in which sounds originating from the front are mistakenly perceived as originating from the rear, or vice versa [18,19]. In addition, localization based only on ITDs is highly susceptible to room reverberation [20], and single-frequency ITD or ILD cues do not adequately represent elevation. By combining ITDs and ILDs over a wide frequency spectrum ranging from approximately 200 Hz to 11 kHz, the proposed biomimetic representation more closely resembles the broadband cue integration performed by the biological auditory system, potentially enabling simultaneous prediction of both horizontal and vertical sound-source locations.

Second, we implemented a biologically inspired “two-step” localization approach that separately classified horizontal and vertical positions. Spectral notches within the mid-frequency range are known to play an important role in vertical localization, especially within the front median plane [21]. As demonstrated in HRTF recordings from the CIPIC Interface Laboratory Database, the notch frequency systematically shifts as the elevation angle changes. Previous studies have shown that combining ILDs with monaural spectral cues improves the resolution of ambiguities associated with time-delay cues [22], thereby facilitating the estimation of both horizontal and vertical sound locations [23]. More recently, full-HRTF localization algorithms have been developed to estimate the locations of multiple sound sources using two microphones [20,24,25] or four microphones [26,27]. These approaches determine source locations by identifying the closest matching HRTF patterns. Inspired by the hierarchical processing of spatial cues in the mammalian auditory pathway, our two-step approach first used binaural HRTF features, consisting primarily of IPDs and ILDs across frequency, to estimate horizontal location. Subsequently, left-ear monaural HRTFs were used to estimate elevation.

Finally, this study examined a full-HRTF localization strategy in which the left and right HRTFs were concatenated into a single feature vector for classifier input. Once localization features had been extracted, a commonly used machine learning classifier, linear discriminant analysis (LDA) [28], was trained using one subset of human recordings and subsequently tested on a different subset obtained from the same database. This framework enabled us to evaluate the extent to which generalized biomimetic spatial-hearing features could transfer across individuals.

In summary, the present study contributes to the development of generalized biomimetic auditory systems capable of human-like spatial hearing without individualized calibration. Such systems may ultimately facilitate the design of next-generation hearing aids, robotic audition platforms and virtual-reality audio systems that emulate the adaptive spatial-processing capabilities of the human auditory system.

## 2. Materials and Methods

### 2.1. Experimental Conditions Using Two HRTF Databases

For the main part of the study, human HRTFs were obtained from the CIPIC Database [16]. In that study, head-related impulse responses (HRIRs) were collected with subjects seated at the center of a 1 m radius hoop, meaning that the distance between subject and speakers was always 1 m. Speakers were placed at various locations along the hoop, with elevation being uniformly sampled from 50 locations (from −45° to 230.625° in steps of 5.625°) and 25 azimuth locations (−80°, −65°, −55°, from −45° to 45° in steps of 5°, 55°, 65° and 80°) forming a 1250-point sphere. The sampling frequency was 44.1 kHz. This database has become a standard benchmark for HRTF-related research, and is widely used in academia and industry for studies of sound localization, binaural synthesis, hearing devices, and personalized spatial audio. With detailed anthropometric measurements, it is possible to study relationships between pinna geometry and acoustic cues such as spectral notches used for elevation perception.

The present study used the HRIRs contained in the CIPIC Database that were obtained from 40 human subjects. For each subject, we chose a smaller subset of recorded locations with azimuth sampled at 12 locations: −65°, −45°, −35°, −25°, −15°, −5°, 5°, 15°, 25°, 35°, 45° and 65°. Elevation was sampled at 10 locations, from −45° to +208.625° with a step of 28.125°, to obtain a subset of 120 locations over the entire sphere. In other words, we decided to reduce the number of classification locations to 10% of the 1250 points in the original database while trying to maintain roughly uniform density on the sphere. To classify 1250 locations, the LDA algorithm would require training data obtained from more human subjects than are available in the existing database. The number of locations we chose (i.e., 120) is already higher than many machine-based localization algorithms [29,30,31,32].

**Figure 1:**
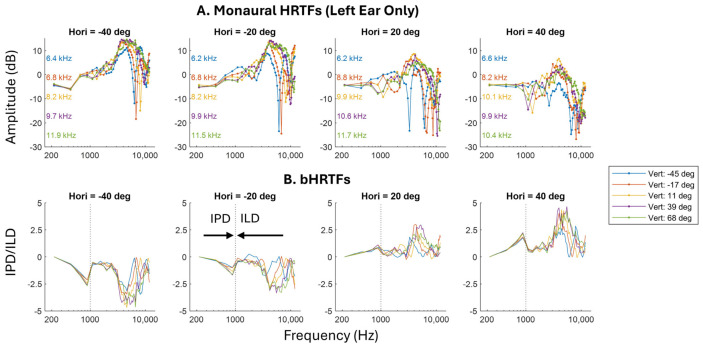
Examples of monaural (**A**) and binaural (**B**) HRTFs for four horizontal locations (columns) and five vertical locations (lines with different colors) using the CIPIC Database. In monaural HRTFs (**A**), the notch frequencies were printed on the left side of each plot. In bHRTFs (**B**), the first four points are IPDs (for frequencies < 1000 Hz). For frequencies higher than 1000 Hz, ILDs are used.

The results obtained with the CIPIC Database will be presented in detail. Figure 1A shows HRTFs obtained with one subject from that database. Each column is a different horizontal angle, and each color-coded trace is a unique vertical angle. The important notch frequencies were printed on the left side of the plot. This mid-frequency notch was derived by finding the minimum dip above 1 kHz. For the four horizontal angles shown here, the notch frequencies varied between 6 and 12 kHz when the sound source increased from −45° to +68° in elevation in each plot. For comparison of performance and consistency, this study also repeated the simulation with another publicly available HRTF database: the Princeton 3D3A Database [17]. In that study, subjects were seated in the chamber in front of a vertical arc, which held 9 loudspeakers. Binaural microphones were inserted into the subject’s ears, and HRIRs were also measured for each loudspeaker. The subject was then rotated in 5° increments horizontally using a computer-controlled turntable upon which the seat was affixed [17]. The sampling frequency of the HRIRs was 32.1 kHz.

In the present study, locations from the 3D3A Database were chosen in an effort to match the CIPIC locations and provide a fair comparison. We were able to choose the same horizontal locations used in the CIPIC Database and got as close as possible to similar locations in elevation, from −57° to +210° with a step of 30°. Thus, a total of 108 locations were chosen over the entire space. This study used the HRIRs obtained from all 38 subjects in our simulations.

Also note that, even at the same elevation angle in terms of a degree, the absolute location of the 3D3A Database is not identical to the CIPIC due to the slightly different setups in the two studies (as explained above). In our simulation study, each of the experiments and paradigms used for the CIPIC Database were repeated for the 3D3A Database but they are not presented in detail.

### 2.2. Classification Based on Three Localization Methods

This study constructed three localization methods as input to the LDA classifier. The same routine of classifications was performed for each feature condition, where the localization plane was split into front, back and entire-field sections to be examined separately. This allowed for a better understanding of where and how errors occurred (such as the front–back confusion) and provided a better overall picture of our localization algorithm performance across different scenarios.

#### 2.2.1. Experiment I (bHRTF): Binaural Spectral Cues Only

In experiment I, localization was carried out using binaural spectral cues alone. First, the HRTFs in the database were expressed as IPDs and ILDs as functions of sound frequency (*f*), referred to as ${Ø}_{IPD}(f)$ and ${Ø}_{ILD}(f)$, respectively. The binaural HRTF (bHRTF) was expressed as a concatenation of IPDs and ILDs:

(1) $$\mathrm{bHRTF}\;=\;\left[\begin{array}{cc} {Ø}_{IPD}\left(f_{1}\right) & {Ø}_{ILD}(f_{2}) \end{array}\right],$$

where ${Ø}_{IPD}(f_{1})$ is the IPD function over 220.5 $\leq f_{1}\leq 882.0\;Hz,$ and ${Ø}_{ILD}(f_{2})$ is the ILD function over 1102.5 $\leq f_{2}\leq 11,907.0\;Hz$. Note that the frequency resolution derived from the HRIRs of the CIPIC Database was 220.5 Hz. Figure 1B shows the corresponding bHRTFs derived from pairs of left-side (Figure 1A) and right-side monaural HRTFs obtained from the CIPIC Database. It can be clearly seen that, for sound sources on the left side, both IPDs and ILDs have entirely negative values (Figure 1B, left two). For sound sources on the right side, both IPDs and ILDs have positive values (Figure 1B, right two). Those behaviors agree with the duplex theory [8]. With a fixed horizontal angle, the curves also vary, but not as much as the variations with horizontal angles, which somewhat explains why the localization acuity is much higher in azimuth than in elevation with both humans and our model predictions. Because the LDA classifier generates the proper weights for each *f* based on the sample distributions across spatial locations, it does not matter what time and intensity are in the same vector.

As mentioned earlier, a subset of 120 vertical or horizontal speaker locations were selected for training in the CIPIC Database and a similar set (i.e., 108) chosen for the 3D3A Database. Having a total of $N_{L}$ bHRTFs, each bHRTF will be assigned with a class (1,2, …,$\;N_{L}$, *L* = 120 or 108). The LDA algorithm was used to classify binaural HRTFs across different locations and individual subjects. A leave-one-out cross-validation (LOOCV) approach [33] was used to provide the training and test samples to the classifier for each session. HRTFs from subjects in the database are split into training and validation sets, according to the LOOCV algorithm. For example, on each trial one of the 40 CIPIC-Database subjects was selected as the test trial, and the rest formed the training pool. A model was trained with the 39 training subjects using the LDA algorithm in MATLAB (MathWorks, R2022b). The test subject was then classified utilizing the trained model. A classification accuracy, $\hat{p}$, was derived from the combined confusion matrix by repeating the procedure for all 40 subjects and combining the confusion matrix results from each trial.

**Figure 2:**
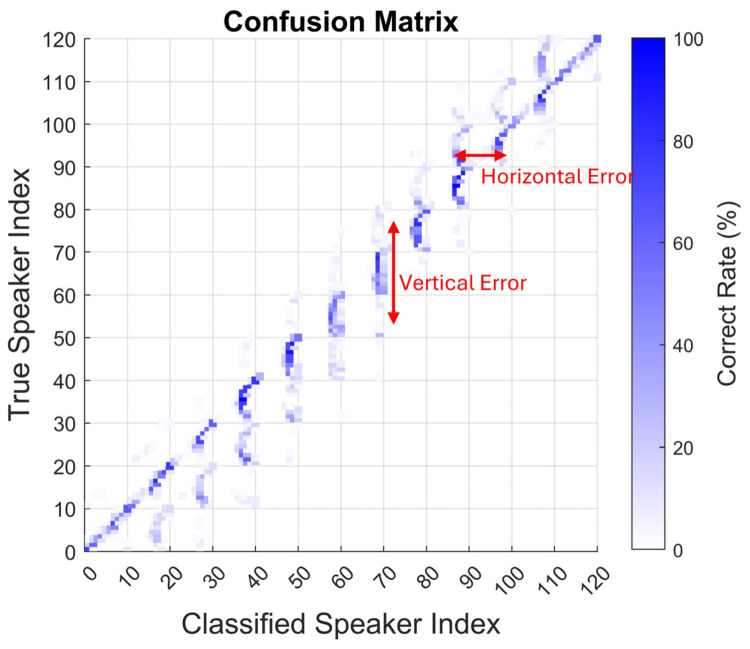
Example of a confusion matrix obtained with bHRTFs for all speaker locations using the CIPIC Database. There are 12 horizontal and 10 vertical locations. Here, for each horizontal location, the speaker index goes through all 10 vertical locations. For example, Speakers 21 to 30 belong to the same horizontal location.

After completion of many classification cycles, for many locations, a confusion matrix can be constructed as a measure of classification accuracy. Figure 2 displays an example of our confusion matrix where bHRTFs for all 120 speaker locations are included. For each horizontal location, the speaker index goes through all 10 vertical locations. For example, Speakers 21 to 30 belong to the same horizontal location. From this, true location identification and errors are determined. A straight vertical line indicates that sound sources have the same horizontal value, but different vertical values were classified as having the same location, e.g., a correct horizontal location but incorrect vertical locations (Figure 2, the red vertical arrow). In contrast, a sudden horizontal jump in the middle of a 10-location group indicates incorrect horizontal classification (Figure 2, the red horizontal arrow). Errors located in the frontal vs. back hemifields are seen in the vertical C-shaped clusters, where a downward right-facing tail is indicative of frontal hemifield errors and an upward left-facing arrowhead indicates back hemifield errors.

In order to view the results more clearly, this study looked at the full-field model compared to frontal and back hemispheres separately. The training and classification procedures using the LDA were the same for each localization method and/or database and therefore will not be repeatedly described.

#### 2.2.2. Experiment II (Two-Step): Separate Monaural and Binaural Cues

In experiment II, classification was carried out in a “two-step” process using dual monaural and binaural spectral cues. First, the horizontal angle was determined using the same bHRTFs described above and the LDA classifier. Next, the vertical angle was determined using only the monaural left-side HRTF (${i.e.,\;HRTF}_{L}$) and the LDA classifier again, since no binaural cue was needed for decoding the spectral changes in elevation. Apart from the fact that different cues were used, the classification procedure using the LDA and the LOOCV remained the same as in Experiment I.

#### 2.2.3. Experiment III (Monaural Pair): Left and Right Monaural HRTFs

In experiment III, classification was carried out using the entire left and right monaural HRTFs. We simply concatenated the two HRTFs (with the frequency range between 220.5 and 11,907.0 Hz) into a single vector.

(2) $$X=\left[\begin{array}{cc} {HRTF}_{L} & {HRTF}_{R} \end{array}\right].$$

X was then used as the input for the LDA classifier to determine both the horizontal and vertical locations/angles.

## 3. Results

We will first present results obtained with the CIPIC Database in detail. Then, we will provide a summary of the results using the 3D3A Database.

### 3.1. Localization Using the bHRTFs

**Figure 3:**
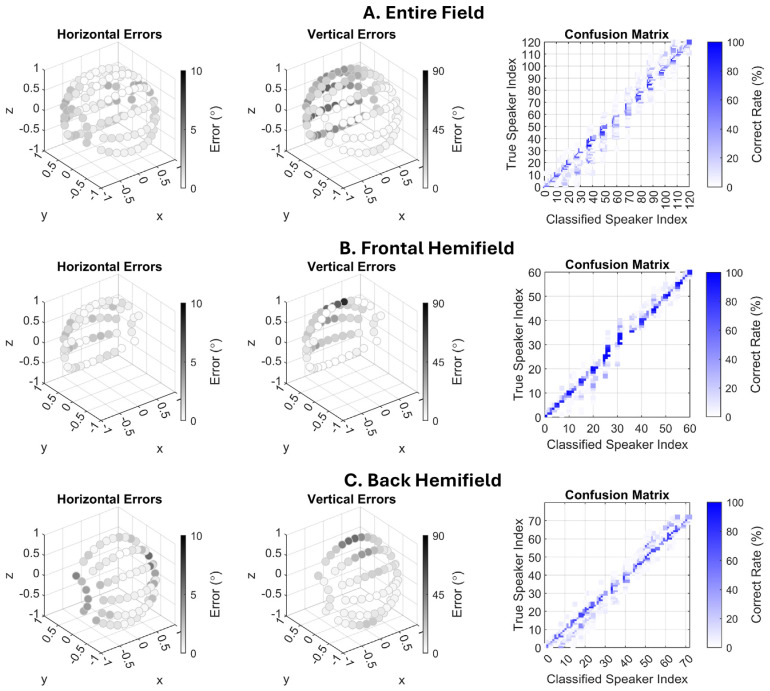
Horizontal and vertical localization errors in the entire space using HRTFs (Condition I; binaural only) obtained from the CIPIC Database. The darkness of each symbol indicates the error value in degrees. (**A**) Errors displayed over the entire field. (**B**) Errors displayed over the frontal hemifield. (**C**) Errors displayed over the back hemifield. The average horizontal and vertical errors for (**A**) were 3.4° and 56.5°, for (**B**), 1.4° and 21.2° and for (**C**), 2.9° and 20.1°, respectively.

Experiment I used the constructed bHRTFs (Equation (1)) for sound-source localization. Figure 3A shows the localization errors over the entire field. Front-to-back hemifields expand from 1 to −1 along the y-axis, with the frontal hemifield extending from 1 to 0 and the back hemifield extending from 0 to −1. The left and right hemifields are expanded along the x-axis from 1 to −1, in the same manner. The color of each symbol represents the error value, with darker colors indicative of higher errors. This is repeated for the frontal and back hemifields in Figure 3B,C, separately.

In general, the horizontal errors were small, which is reasonable given that we used the binaural cues here. For the vertical errors, the majority of the errors in the experiments occurred in the frontal hemifield. This may have been caused by the front–back confusion in the model, creating a larger localization error. In this case, we found a large number of frontal speaker locations being confused with locations in the back hemifield.

We may gain more insights into where errors occur over the entire field through investigating the confusion matrix (Figure 3, rightmost). In particular, it is often observed that speakers with the same horizontal location but different vertical locations are classified as the same speaker, e.g., speaker Indexes 19, 29, 39, 89 and 108. We see these are frontal errors, as indicated by the right-facing downward tails of the vertical C-clusters at the above-mentioned classified speaker locations. Indeed, Experiment I had an average horizontal error of 3.4° and an averaged vertical error of 56.5°.

To measure the classification performance free of the front–back confusion, the two hemifields were examined separately. First, we performed the classification in the frontal hemifield alone (Figure 3B). The performance was notably improved. The average horizontal error was reduced to 1.4° and the average vertical error was reduced to 21.2°. Figure 3C shows the results in the back hemifield alone. Again, the averaged horizontal error was reduced to 2.9° and the averaged vertical error was reduced to 20.1°.

Overall, we believe that the front–back confusion was a significant factor in the classification error over the entire field (Figure 3A).

### 3.2. The “Two-Step” Localization

Next, we applied a “two-step” approach by determining the horizontal locations using the bHRTFs, followed by determining the vertical locations using the left-side monaural HRTF.

**Figure 4:**
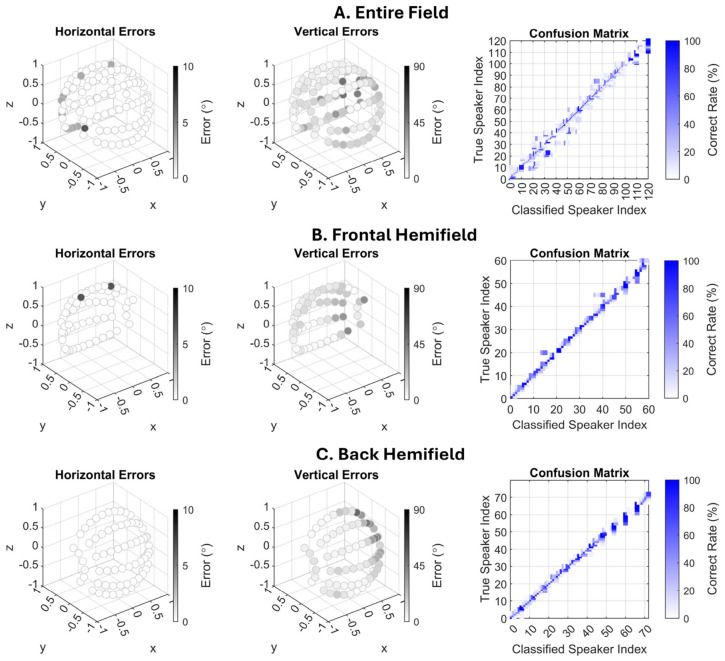
Horizontal and vertical localization errors in the entire space and separated by front and back hemifields using dual HRTFs obtained from the CIPIC Database (Condition II). Everything is in the same format as Figure 3. The average horizontal and vertical errors for (**A**) were 1.0° and 30.4°, for (**B**), 0.3° and 14.3° and for (**C**), 0.0° and 21.6°, respectively.

Figure 4 has the same format as Figure 3. For the horizontal errors (leftmost column), using the two-step approach notably reduced the errors (leftmost). For the vertical errors, the worst ones occurred in the frontal hemifield, indicated by darker symbols in the plot (Figure 4A, middle). This is explained again by the front–back confusion in the model, where in this case we see a large number of front–back speaker locations confused for being located in the back/frontal hemifield, respectively. Looking at the confusion matrix, we mostly identify vertical errors indicated by the C-shaped clusters. The averaged horizontal error was 1.0° and the averaged vertical error was 30.4°.

Next, we examined both frontal and back hemifields separately. In the frontal hemifield, we see very few errors overall, given that there are very few darker symbols present in the plot (Figure 4B, leftmost). This is also confirmed in the confusion matrix where there are very few errors shown (Figure 4B, rightmost). Our best overall localization performance thus far is obtained here, with an averaged horizontal error of 0.3° and an averaged vertical error of 14.3°.

In the back hemifield, there was no error in the horizontal localization performance (Figure 4C, leftmost). We see that the majority of large vertical errors appear on the back-right side (Figure 4C, middle). The average horizontal error was 0.0° and the average vertical error was 21.6° in the back hemifield under the conditions used for experiment II.

### 3.3. The “Monaural Pair”

**Figure 5:**
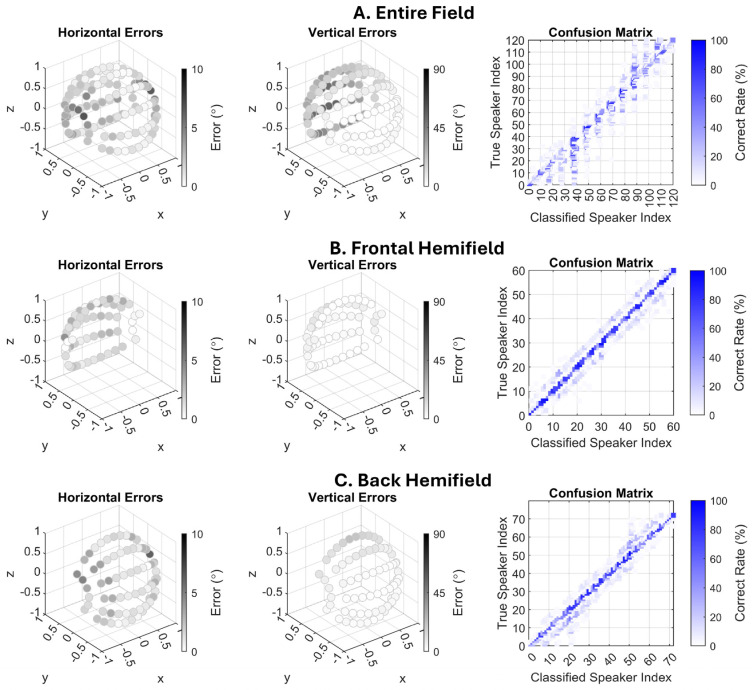
Horizontal and vertical localization errors in the entire space and separated by front and back hemifields using left and right entire HRTFs obtained from the CIPIC Database (Condition III). Everything is in the same format as Figure 3. The average horizontal and vertical errors for (**A**) were 5.6° and 54.6°, for (**B**), 2.4° and 3.6° and for (**C**), 3.3° and 8.0°, respectively.

As mentioned earlier, the localization feature used here is a concatenation of the left and right HRTFs for each sound location (Equation (2)). Looking at the entire field (Figure 5A) first, we obtained an average horizontal error of 5.6° and an average vertical error of 54.6°. The larger errors in the vertical space are indicated by the darker symbols in the figure (middle), where most occur in the frontal hemifield due to front–back confusion there.

Looking at the fontal hemifield in Figure 5B, we obtained an averaged horizontal error of 2.4° and an averaged vertical error of 3.6°. For the back hemifield in Figure 5C, we obtained an average horizontal error of 3.3° and an average vertical error of 8.0°.

### 3.4. Summary of Results Using the CIPIC Database

**Figure 6:**
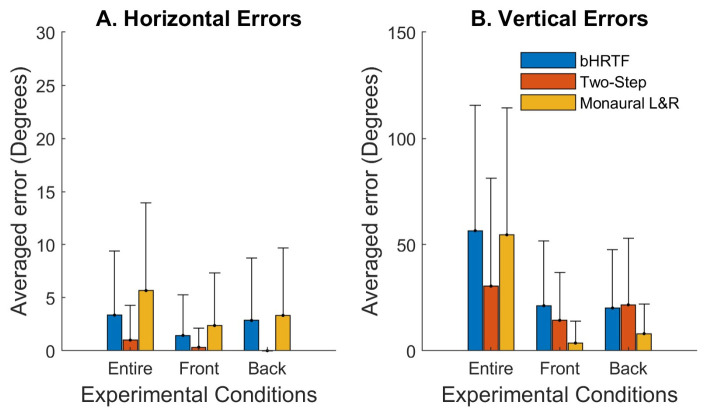
Localization errors in azimuth (**A**) and elevation (**B**) using the CIPIC Database for the entire field, the frontal hemifield, and the back hemifield, respectively.

The overall error values along with mean and standard deviation for Experiments I, II and III are plotted in Figure 6. Because the values span a large range, we also put them in Table 1. In general, the horizontal errors were much smaller than the vertical errors. The two-step approach generated horizontal errors that were close to 0. For vertical errors, the monaural-pair approach yielded very small errors for the front and back hemifields, but not for the entire field, presumably due to front–back confusion.

Next, we performed a two-way Analysis of Variance (ANOVA) test to determine the influence of the “experimental condition” (i.e., bHRTFs, two-step, and monaural pair) and the “localization space” (i.e., entire, frontal, and back). Table 2 summarizes the ANOVA results. We would like to point out that the large numbers of “Sum of Squares” are due to the fact that we are adding 30,240 squared error values over all conditions, spaces, locations and human subjects. When examining the Mean Square of the ANOVA error for the horizontal results, $\sqrt{29}=5.4^{{}^{\circ}}$. This number indicates the variability in the data not accounted for by the two variables, condition and space, and is also on par with the horizontal errors we achieved in Figure 6A. Similarly, in the vertical localization table, the Mean Square of error was $\sqrt{1840}=42.9^{{}^{\circ}}$, which was also on par with the vertical errors in Figure 6B.

For both horizontal and vertical localizations, the two variables, condition and space, were highly significant (*p* close to 0). For horizontal performance, condition had a higher *F* value than space. For vertical performance, space had a higher *F* value than condition.

### 3.5. Performance Using the 3D3A Database

**Figure 7:**
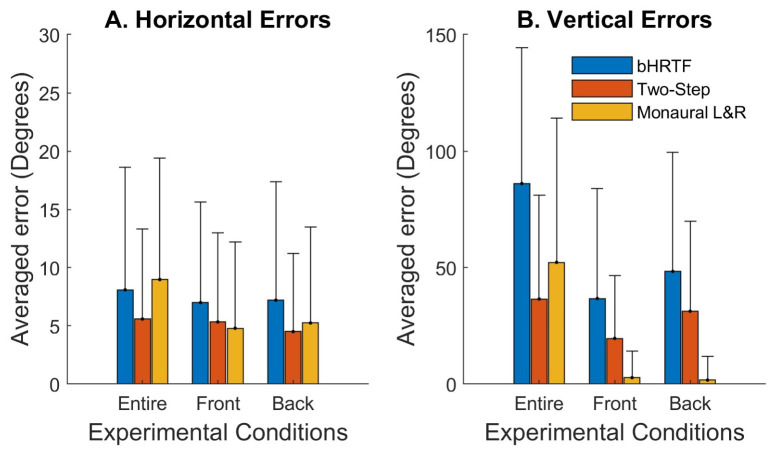
Error results for vertical and horizontal errors using the 3D3A Database in the same format as Figure 6.

To examine if our findings hold for other HRTF databases, we then tested the 3D3A Database [17] and repeated each of our previously defined conditions. The general observation using the 3D3A Database (Figure 7) was highly similar to what was observed with the CIPIC Database (Figure 6), except that errors were larger. The Pearson’s correlation coefficient for the horizontal errors between the two databases was 0.74, and for the vertical errors it was 0.89. Both were highly significant.

The “two-step” approach was the best method for both databases, yielding 1.0° and 30.4° as horizontal and vertical errors for the entire field using the CIPIC Database, respectively. It again showed 5.6° and 36.5° as horizontal and vertical errors for the entire field using the 3D3A Database, respectively.

### 3.6. The Gender Effect

Both HRTF databases provided anthropometry data associated with each human subject. The CIPIC database also provided the gender information, including 26 male subjects, 13 female subjects, and one unidentified subject. In all of the above simulations, the LOOCV method combined both genders when training the classifier and predicting for the test subjects. Since the gender data has distinct subsets, we can easily examine the gender effect by separating the training and testing groups based on gender. It is more difficult to examine other anthropometry data that is typically continuous.

**Figure 8:**
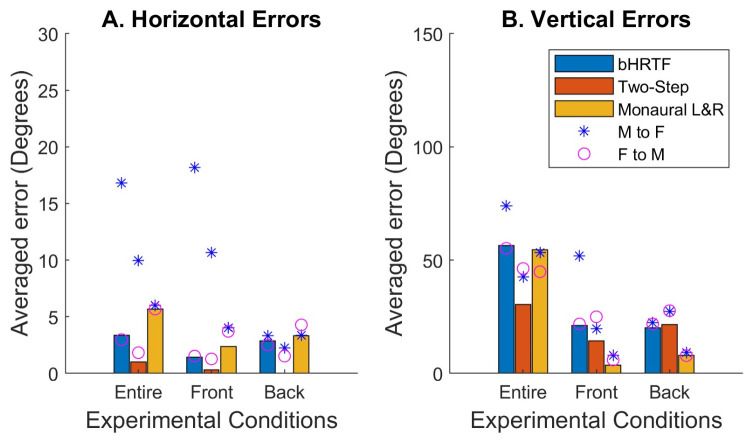
Localization errors obtained with the CIPIC Database re-plotted from Figure 6, with additions of gender-separated predictions. Blue asterisks represent LDA classifiers trained with male subjects used for the predictions of female subjects (M to F). Pink circles represent classifiers trained with female subjects used for the predictions of male subjects (F to M).

Here, we re-ran the three algorithms with males and females separated. Specifically, we first trained the LDA classifier using the 26 males’ HRTFs and made predictions for the 13 females. Figure 8A (blue asterisks) shows the performance for the horizontal errors, which were considerably larger than the gender-mixed data for the entire and front hemifields using the bHRTF and the two-step methods. For the back hemifield, horizontal errors increased from 0° to 2.2° using the two-step methods. The monaural-pair methods also showed an increase for the front hemifield from 2.37° to 4.04°, but not for the entire or back conditions.

Next, we trained the LDA classifier using the 13 females’ HRTFs and made predictions for the 26 males. Figure 8A (pink circles) shows the performance for the horizontal errors. Although the female classifier’s performance was also worse in some cases, it did not exhibit errors as large as the male classifier.

Figure 8B shows the performance of the vertical errors. Again, the male classifier showed the largest error in certain conditions.

## 4. Discussion

### 4.1. Motivation for Robotics Sound Localization

In everyday interactions, we find ourselves in environments where sounds come from multiple sources and locations. Although human listeners can localize more than one sound source concurrently [34,35], this is not the case for the hearing impaired. Standard hearing devices and aids, including modern cochlear implants, do not effectively restore a normal level of sound source localization for hearing-impaired listeners [5]. From a biomimetic perspective, understanding and replicating the biological mechanisms underlying spatial hearing is therefore critical for the development of next-generation hearing-assistive technologies.

The end goal of our study is to provide hearing-aid listeners with instant locations of nearby sound and the ability to selectively amplify/attenuate certain sound sources. Two steps are involved in this process: (1) spatial features are identified for each sound source, and (2) speech is segregated from other sound based on the identified features [1,36,37,38]. An ideal hearing-aid application should provide a detailed spatial map of all surrounding sources so that the user can select the sources to be attenuated or amplified. Therefore, our main focus in this study was to investigate the issue of variability in HRTF recordings when applied to a large user base. Positive results seen through this study serve as an initial step (i.e., sound localization) toward achieving this goal and provide insight into areas for future improvement moving forward. In an accompanying study [38], we examined an automatic speech-segregation algorithm that can be integrated with the algorithms presented here.

Robotic audition and hearing-assistive technologies have attempted to emulate the biological processing of HRTFs to achieve human-like sound localization. However, a major challenge is the substantial inter-subject variability observed in HRTFs due to differences in head size, pinna morphology, and torso geometry [39,40,41,42,43,44,45]. Previous studies have suggested that hearing devices may require adaptation to the individual user’s ear acoustics in order to achieve accurate spatial perception [46,47]. Ideally, individualized HRTFs would be measured for every user of a spatial-hearing device; however, HRTF acquisition is time-consuming, technically demanding, and impractical for widespread clinical or consumer use.

Recent studies have therefore investigated whether individualized HRTFs can be predicted computationally from anthropometric measurements or anatomical reconstructions using deep learning approaches. For example, neural network-based frameworks have been developed to estimate individualized HRTFs from three-dimensional head meshes and anthropometric features [45,47,48]. Other studies have demonstrated that HRTF interpolation and reconstruction can be achieved using neural-process and meta-learning approaches, suggesting that generalized spatial-hearing representations may transfer across users despite anatomical variability [49]. Therefore, a key question in biomimetic auditory engineering is whether generalized spatial-hearing principles can be extracted from population-level HRTF datasets and transferred to new individuals without requiring subject-specific calibration. If robust spatial features can be learned from existing HRTF databases and generalized across users despite anatomical variability, future biomimetic hearing systems may achieve accurate localization without individualized measurements. Such an approach would significantly simplify the implementation of next-generation hearing aids, augmented-reality audio systems, and robotic auditory platforms.

Because real-life sound often has a “sparseness property” in the time–frequency spectrogram, blind-source separation techniques can then be used to derive the HRTFs of ongoing sound [24,38,50]. Previous studies have compared the derived HRTFs from the time–frequency clusters with a pre-existing HRTF database that is likely not matched well to the listener. This is an issue due to the variability in the HRTFs with individual human subjects and its effect on the localization accuracy. In addition, because localization in elevation mostly relies on monaural spectral cues, the effectiveness of using binaural spectral cues (the difference between the two ears as a function of frequency) to localize sources in elevation needs further examination.

### 4.2. The Three Sound-Source Localization Methods

Previous studies on robotics sound localization or spatial-speech segregation mostly used the original monaural HRTF pairs [24,25,51,52]. Although the present study also included this method in experiment III, our study constructed two additional algorithms—the bHRTFs and two-step methods. Depending on the localization conditions, such as whether one used the entire field vs. front or back hemifield, whether horizontal or vertical locational was the focus, and the speed or simplicity of the algorithm, the three methods exhibited advantages in different aspects.

In the first bHRTF method, we used only binaural spectral cues. This is the simplest method with the fastest computational speed among the three. We found that spatial sound-source detections were most successful in the frontal hemifield, and that horizontal locations were more accurately detected than vertical locations, as expected. The lowest error rate was seen when examining only the frontal hemifield, where the averaged horizontal error was 1.4° and the averaged vertical error was 21.2° using the CIPIC Database (Figure 6A,B, blue). Comparing this performance to that found over the whole field of 3.4° and 56.5° for horizontal and vertical average errors, respectively, and average horizontal and vertical errors in the back hemifield of 2.9° and 20.1° respectively, we conclude that bHRTFs are always a good method for horizontal localization and that they can also detect the vertical locations to some degree, but suffer from large front-and-back confusion.

When switching to the 3D3A Database (Figure 6A,B, blue), the general trend remained, although errors in both azimuth and elevation increased. The vertical error for the entire field was especially large, presumably due to front-and-back confusion. We should point out that, although the bHRTF method suffers from large front-and-back confusion, it does provide a certain ability to detect the vertical location because it is a frequency-dependent feature covering a large frequency range, thus providing certain spectral features. This is different from using ITDs or ILDs at a single frequency.

Overall, while each experimental method was able to correctly detect sound-source locations with various success rates, the two-step approach using both binaural and monaural localization cues in a sequential process detected the horizontal and the vertical locations separately and yielded the best localization accuracy in azimuth for both databases (Figure 6A and Figure 7A, orange). This finding was not surprising given that binaural cues are best for localization in azimuth and monaural spectral cues are best for localization in elevation. The averaged horizontal error was 1.0° and the averaged vertical error was 30.4° for the entire field using the CIPIC database. We also observed similar improvements in the back hemifield, where the horizontal and vertical averaged errors were greatly reduced to 0.0° and 21.6°, respectively. Finally, in the frontal hemifield we saw a significant improvement in elevation with a horizontal average error of only 0.3° and a vertical average error of 14.3°.

Examining front and back hemifields separately under all experimental conditions, we saw that front–back confusion is a big source of error in vertical sound source localization. In particular, when separating the front and back hemifields, we often observed a significant improvement in error values for vertical localizations. Out of the three methods, the two-step approach was the most “confusion-resistant”. Note that only the left-side monaural HRTF was used in the two-step approach. When examining either the front or the back hemifield, the concatenated monaural-pair approach (Figure 6B and Figure 7B, yellow) provided the lowest error values for both databases. However, this method somehow suffers more from the front-and-back confusion than the two-step approach for both databases.

In practice, the choice of localization methods depends on the focus of the application. Human listeners are usually involved in situations where only the horizontal locations of the surrounding sound sources matter, such as in a restaurant setting. In that case, the two-step method is the best choice since it provides the best horizontal performance. The monaural-pair method generates the smallest errors in elevation when only the front or back hemifield is considered but does not do well for the entire field; thus, it is not the ideal choice. The bHRTF method will be the best choice if speed and computational cost are the priorities.

### 4.3. Findings on Par with Human Vertical Sound Source Localization Capabilities

Our best performance is on par with that of a non-hearing-impaired human listener, where previously mentioned human-psychophysical studies showed localization errors of ~1.0° in the azimuth and ~10.0° in elevation [19]. This means that, in general, it will be ten times more difficult to detect vertical sound source localizations versus horizontal localizations in humans, let alone machines. Therefore, it was quite an achievement for our algorithm to detect sound source localizations at this accuracy. It is also frequently observed that sound sources are detected more accurately in the frontal hemifield than in the back, which is in agreement with the human ability to localize sound sources, in which it is easier to locate sounds in the frontal hemifield versus the back [53].

Regarding the large errors in elevation, especially for the entire field where the front–back confusion exists, one possible explanation is that we used HRTFs obtained from some human subjects to predict sound locations for other subjects. Because ITDs and ILDs only depend on the head size and shape, individual variations are typically limited. That explains why the horizontal-localization errors were very small. In contrast, localization in elevation relies on the spectral shape of the HRTFs. HRTFs are highly sensitive to the bony structures and morphology of the pinnae [14], which can vary dramatically across humans. This is probably where our generalization failed to work accurately to the greatest degree.

Furthermore, our main goal was to determine if a database of HRTF filters, such as the CIPIC Database, could be used for sound-source localization for novice users. Given that, in the LOOCV method, we used one member of the CIPIC Database for testing and the rest to form the training pool, alternating through each member of the database, this indicates that sound localizations for new users can in fact be detected using pre-recorded HRTF signals. This is especially important to the success of this study, as due to the time, attention to detail and equipment needed for HRTF filter collection, the need for individualized HRTF signals or for more custom signal-collection processes would be a large setback reducing the feasibility of this technology.

While the results of this study show promise in overcoming the challenge of sound source localization for hearing-impaired listeners, there is still more work to be done in terms of developing a solution that fully meets listener’s needs. Future work will focus on continuing to improve our algorithm and working to overcome the front–back confusion challenge. We also plan to introduce room reverberations in the future, where reverberations will be simulated with an auralization software (CATT-Acoustic, v9.1) and applied to the loudspeaker sound. Tests will be applied to the obtained angular errors to examine the influences of sound type and room reverberation on the localization accuracy. This will help us to better understand the performance of this system in real-world situations and environments where reverberations will arise.

### 4.4. Individual Variability

HRTFs can vary greatly among human subjects [39,40,41,42,43,44]. Denk et al. [46] recorded individualized HRTFs in the ear drum of 16 human subjects. They used an auditory model and virtual acoustic space to evaluate spectral deviations between the computed target and listening with the open ear. Their results indicate that individual correction functions can profoundly reduce spectral deviations to open-ear listening as compared with generic correction functions. Ellinson and Gannot [52] found out that, by using individualized HRTFs, they were able to separate speech sound from interference in an anechoic condition.

Although anthropometry data has been provided on the head and pinna parameters, our overall classification accuracy cannot be easily correlated to those continuous variables. We were able to examine one factor of the individuality, which is the gender. We first trained the classifier using the male’s HRTFs and made predictions for the female’s locations. If there were no gender effects, localization errors should have been on par with what we previously achieved when genders were mixed. However, considerably larger errors can occur under certain experimental conditions. In contrast, when we trained the classifier using the female’s HRTFs and made predictions regarding the male’s locations, the performance was generally on par with the mixed-gender results. A possible explanation is that the 26 males’ HRTFs exhibited large variability within them to the extent that the LDA classifier could not be sufficiently trained under certain experimental conditions. Therefore, the poorly trained classifier will likely make even worse predictions for females’ locations. On the contrary, the classifier must have been sufficiently trained using the 13 females’ HRTFs to generate a reasonable performance for the males’ locations. Unfortunately, we were only able to examine the gender effect for the CIPIC database, as the 3D3A database did not provide gender information. Simulations with more HRTF databases are needed to draw firm conclusions.

## 5. Conclusions

Even with the variabilities created by gender, overall, this study found that using the HRTFs obtained from some human subjects to predict sound locations for other subjects yielded satisfactory localization performance and acceptable errors, especially for the azimuth. The averaged localization errors over the entire field were 1.0° and 30.4° in horizontal and vertical planes, respectively, using the CIPIC Database. Furthermore, with separation between the front and back hemifields, localization was improved by up to 50% with averaged errors in the frontal hemifield of 0.3° and 14.3° for horizontal and vertical errors, respectively. Using two databases that were obtained separately by two different research groups, our results showed highly similar patterns with the three constructed localization features. Grouping the training and testing HRTFs based on gender indicated that performance may be highly affected by individuals or subsets of human groups.

## Figures and Tables

**Table 1:** Averaged horizontal and vertical error results using the CIPIC Database. STD—standard deviations.

	Experiment I: bHRTF			Experiment II: Two-Step			Experiment III: Monaural Pair		
	Entire	Front	Back	Entire	Front	Back	Entire	Front	Back
Horizontal Errors	3.4°	1.4°	2.9°	1.0°	0.3°	0.0°	5.6°	2.4°	3.3°
Vertical Errors	56.5°	21.2°	20.1°	30.4 °	14.3°	21.6°	54.6°	3.6°	8.0°
Horizontal STD	6.1°	3.8°	5.9°	3.3°	1.8°	0.0°	8.3°	5.0°	6.4°
Vertical STD	59.2°	30.4°	27.4°	50.9°	22.6°	31.4°	59.8°	10.3°	13.9°

**Table 2:** Analysis of Variance (ANOVA) using the CIPIC Database. * Interactions between two variables.

**Horizontal ANOVA Results**					
**Source**	**Sum Sq.**	***df***	**Mean Sq.**	**F**	**Prob > F**
Condition	5.28 × 10^4^	2	2.64 × 10^4^	911.9	0
Space	2.08 × 10^4^	2	1.04 × 10^4^	358.4	0
Condition*Space	7.51 × 10^3^	4	1.88 × 10^3^	64.8	0
Error	8.75 × 10^5^	3.02 × 10^4^	29.0		
Total	9.72 × 10^5^	3.02 × 10^4^			
**Vertical ANOVA Results**					
Condition	6.71 × 10^5^	2	3.36 × 10^5^	182.5	0
Space	81.9 × 10^5^	2	40.9 × 10^5^	2.22 × 10^3^	0
Condition*Space	11.0 × 10^5^	4	2.74 × 10^5^	148.9	0
Error	55.6 × 10^6^	3.02 × 10^4^	1.84 × 10^3^		
Total	65.7 × 10^6^	3.02 × 10^4^			

## Data Availability

MATLAB codes and simulation data can be found at the following online data repository. https://dataverse.harvard.edu/dataverse/hrtfgai (accessed on 28 June 2026).

## Abbreviations

The following abbreviations are used in this manuscript:

CIPIC	Center for Imaging Processing and Integrated Computing.
HRTF	Head-related transfer functions.
LOOCV	Leave-one-out cross-validation.
LDA	Linear discriminant analysis.
ITD	Interaural time difference.
ILD	Interaural level difference.
IPD	Interaural phase difference.
